# Combined Model for the Diagnosis of Hepatocellular Carcinoma: A Pilot Study Comparing the Liver to Spleen Volume Ratio and Liver Vein to Cava Attenuation

**DOI:** 10.3390/jcm14124306

**Published:** 2025-06-17

**Authors:** Ludovico Abenavoli, Giuseppe Guido Maria Scarlata, Maria Luisa Gambardella, Caterina Battaglia, Massimo Borelli, Francesco Manti, Domenico Laganà

**Affiliations:** 1 Department of Health Sciences, University “Magna Graecia”, 88100 Catanzaro, Italy; giuseppeguidomaria.scarlata@unicz.it (G.G.M.S.); marialuisa.gambardella@studenti.unicz.it (M.L.G.); 2 Department of Experimental and Clinical Medicine, University “Magna Græcia”, Viale Europa, 88100 Catanzaro, Italy; caterina.battaglia@unicz.it (C.B.); domenico.lagana@unicz.it (D.L.); 3 UMG School of PhD Programmes “Life Sciences and Technologies”, University “Magna Graecia”, 88100 Catanzaro, Italy; massimo.borelli@unicz.it; 4 Radiology Unit, Renato Dulbecco University Hospital, 88100 Catanzaro, Italy; mantifra@unicz.it

**Keywords:** liver cirrhosis, imaging, diagnosis, biomarkers, scores, chronic liver disease

## Abstract

**Background/Objectives:** Hepatocellular carcinoma (HCC) is a major cause of cancer-related mortality and often develops in the context of liver cirrhosis (LC). Its detection remains a clinical challenge, particularly with limited sensitivity of the current serum biomarkers and qualitative imaging tools. The aim of this pilot study is to evaluate the application of a combined model based on the use of Liver to Spleen Volume Ratio (LSVR), a score of regional liver remodeling, and Liver Vein to Cava Attenuation (LVCA), a computed tomography (CT)-based perfusion-related parameter, to diagnose HCC in patients with LC. **Methods**: In this observational retrospective pilot study, 36 patients with LC, with or without HCC, were enrolled from a single tertiary care center between 2021 and 2024. Demographic, clinical, biochemical, and imaging data were collected. LSVR and LVCA were calculated from contrast-enhanced CT scans. Predictors of HCC were assessed using conditional inference trees and multivariate logistic regression. Model performance was evaluated using the area under the receiver operating characteristic curve (AUC). A *p*-value < 0.05 was considered statistically significant. **Results**: LVCA and LSVR levels were significantly higher in the HCC group (*p* < 0.001). In multivariate analysis, LVCA was significantly associated with HCC onset (Odds Ratio = 2.88, *p* = 0.0075). The final model incorporating both LVCA and LSVR achieved excellent discrimination (AUC = 0.967), with 91% sensitivity and 88% specificity. The combined model outperformed LSVR alone (*p* = 0.030), though not LVCA alone. **Conclusions**: Our pilot study suggests the utility of LVCA and LSVR as potential non-invasive imaging tools for HCC diagnosis. External validation in multicenter cohorts and longitudinal studies assessing the temporal evolution of LSVR and LVCA are necessary to better evaluate their application in clinical practice.

## 1. Introduction

Hepatocellular carcinoma (HCC) is the most common primary malignancy of the liver and represents a major global health burden. It typically arises in the context of chronic liver diseases and advances through a well-characterized sequence from liver injury to fibrosis, cirrhosis, and ultimately neoplastic transformation [1]. The pathogenesis of HCC involves a complex interplay of genetic, epigenetic, and inflammatory mechanisms, often driven by underlying etiologies, such as chronic hepatitis B (HBV) or C (HCV) virus infection, alcohol-related liver disease, or non-alcoholic steatohepatitis [2]. Globally, HCC accounts for over 80% of primary liver cancers and is the third leading cause of cancer-related death, with a five-year survival rate of less than 20% in most populations [3]. Its incidence continues to rise in parallel with increasing rates of metabolic-associated steatotic liver disease, further underscoring the need for effective strategies for early detection and risk stratification [4]. Imaging plays a central role in the diagnosis of HCC, particularly in patients with known liver cirrhosis (LC). Ultrasonography is widely used as a first-line surveillance tool due to its non-invasiveness, ubiquitousness, and relatively low cost. However, its sensitivity is limited, especially in detecting small or deep hepatic lesions [5]. Magnetic resonance imaging (MRI) offers excellent soft tissue contrast and high diagnostic accuracy, particularly with hepatobiliary contrast agents, yet its availability, cost, and contraindications limit widespread use [6]. Computed tomography (CT) remains a cornerstone in HCC diagnosis, offering a high spatial resolution and rapid acquisition. However, inter-observer variability and challenges in distinguishing benign regenerative nodules from early HCC highlight the need for quantitative, standardized imaging biomarkers [7]. Despite the advances in imaging, non-invasive serum biomarkers for HCC, such as alpha-fetoprotein (AFP), lack sufficient sensitivity and specificity to be used as standalone diagnostic tool [8]. Emerging laboratory parameters, including des-γ-carboxy prothrombin (DCP) and lens culinaris agglutinin-reactive AFP (AFP-L3), have shown promise, but their performance varies across populations and disease stages [9]. Currently, no single non-invasive biomarker can predict its development with high accuracy. The limitations of these latter methods emphasize the ongoing need for novel diagnostic tools that can be seamlessly integrated into clinical workflows, particularly in high-risk populations [10,11,12]. Timely diagnosis of HCC is crucial, as it directly influences treatment options and patient outcomes [13] Accordingly, there is a growing interest in quantitative imaging features and composite imaging-based scores, which may enhance diagnostic accuracy and serve as potential predictors of HCC. These include morphological and perfusion-based parameters derived from routine imaging modalities, which could improve risk stratification and decision-making in at-risk patients [14]. Specifically, the Liver to Spleen Volume Ratio (LSVR) reflects regional liver remodeling. It has emerged as a valuable imaging biomarker in chronic liver disease, particularly for assessing architectural distortion and portal hypertension. In particular, LSVR can support non-invasive staging and risk stratification in clinical practice [15]. Also, the Liver Vein to Cava Attenuation (LVCA) ratio, a perfusion-related parameter derived from contrast-enhanced CT, provides indirect information about hepatic hemodynamics and microvascular changes. This diagnostic tool has shown potential in identifying changes in portal flow and sinusoidal perfusion associated with advanced liver injury [16]. The present pilot study aims to evaluate the application of a combined model, based on the use of LSVR and LVCA, to diagnose HCC in patients with LC.

## 2. Materials and Methods

### 2.1. Study Design

This observational, retrospective, pilot study included 36 patients who were evaluated at the “Renato Dulbecco” University Hospital in Catanzaro, Italy, between 1 January 2021 and 31 December 2024. According to the international guidelines, cirrhotic patients underwent regular follow-up programs, which included hepatic ultrasound and AFP blood-level assessments every six months. In addition, the subjects with detected suspicious nodules or a Liver Imaging Reporting and Data System score in the range of 4–5, underwent contrast-enhanced abdominal CT scans to evaluate the presence of HCC [17]. Eligibility criteria were as follows: (i) age ≥ 18 years; (ii) confirmed diagnosis of LC based on a combination of clinical findings, laboratory results, imaging findings, and/or histopathological evaluation; and (iii) diagnosis of HCC confirmed through CT [18,19]. Patients who were pregnant or breastfeeding at the time of the evaluation were excluded from this study.

### 2.2. Data Collection

Patient data were extracted from electronic medical records and included demographic information (age and gender), underlying etiology of LC, number of cirrhosis-related complications, presence of esophageal or gastric varices, grading of esophageal varices, presence of hepatorenal syndrome, portal hypertensive gastropathy, portal vein ectasia or thrombosis, and splenomegaly [19,20,21]. Tumor-related variables included the focality of HCC (monofocal or multifocal), size of the hepatic nodules, total liver volume, spleen volume, right hepatic lobe diameter, and spleen diameter [22]. In addition, the following laboratory parameters were assessed: serum albumin, alkaline phosphatase (ALP), aspartate aminotransferase (AST), alanine aminotransferase (ALT), activated partial thromboplastin time (aPTT), serum creatinine, fibrinogen, γ-glutamyl transferase (GGT), AFP, international normalized ratio (INR), prothrombin time (PT), platelet count, serum potassium, sodium, and total bilirubin levels. At the same time, the Child–Pugh, Model for End-Stage Liver Disease (MELD), and MELD-Na scores were calculated for each patient [23].

### 2.3. LSVR and LVCA Calculations

**Figure 1:**
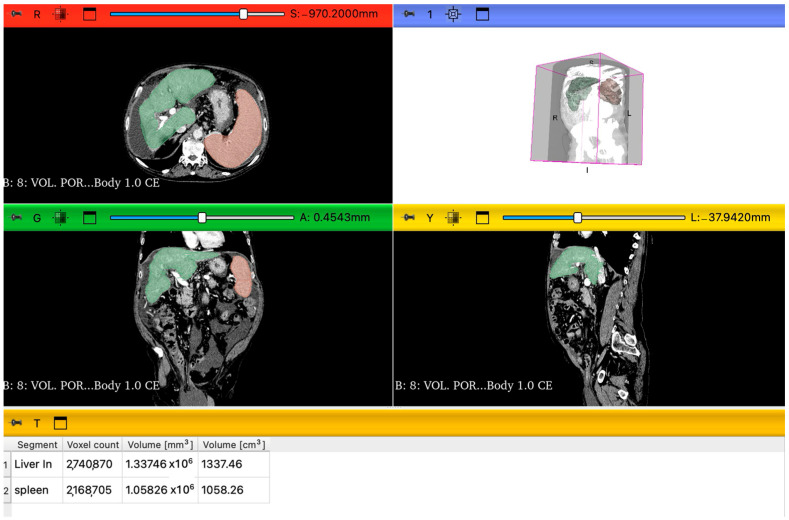
Representation of 3D slicer segmentations of liver and spleen parenchyma in the axial view, sagittal view, and coronal view. Green is the color of the liver, and orange is the color of the spleen. Under the visual segmentation, there is the analysis of images using the extension “Segment statistics” and the volume values (cm^3^).

The images were obtained with CT scan (Aquilon 64, Toshiba, Mezzago, Italy) using portal venous-phase imaging performed at 76 s after the intravenous administration of the iodine contrast agent Iomeron 400 (Bracco S.p.a., Milan, Italy). One-millimeter axial slices were reconstructed with an increment of 1 mm in a liver parenchyma window using a vendor-specific iterative reconstruction algorithm. The evaluations were performed by two radiologists, one with more than 15 years of experience. Portal venous phase CT images were processed using 3D Slicer (version 5.8.2), an open source software for medical image processing and visualization. Specifically, we utilized Digital Imaging and Communications in Medicine images to facilitate the analysis. The liver and spleen volume measurements were performed first using the module “RVX Liver Segmentation” and then, for the number values, the “Segment Statistics” extension (Figure 1). The LSVR was calculated as liver volume divided by spleen volume [24]. The LVCA is an ordinal scoring system that evaluates the density of the liver veins located 1 cm before their confluence, in comparison to the inferior vena cava 1 cm below the confluence point. The evaluation is primarily visual, comparing the appearance of the liver veins and the inferior vena cava. When their attenuation appeared similar, regions of interest were placed in the liver veins and in the inferior vena cava at the specified locations for precise measurement. If the average density of the three liver veins was within ±20 Hounsfield Units of the vena cava’s density, the veins were categorized as isoattenuating (LVCA score of 2). If the difference exceeded this range, the liver veins were classified as either hyperattenuating (score of 1) or hypoattenuating (score of 3). If the liver veins showed no contrast enhancement at all, they received an LVCA score of 4. The LVCA was assessed by comparing the attenuation of the liver veins to the inferior vena cava, as described previously [25].

### 2.4. Statistical Analysis

Continuous variables were presented as mean ± standard deviation (SD), while categorical variables were expressed as absolute frequencies and corresponding percentages. Normality of distribution for continuous data was assessed using the Shapiro–Wilk test. Depending on the data distribution, comparisons between groups were performed using either the independent samples *t*-test or the Mann–Whitney U test. Categorical data were analyzed using Fisher’s exact test. To investigate HCC predictors, we employed both exploratory recursive conditional tree regression and traditional generalized linear modeling approaches. Conditional inference trees, as implemented in the party package [26,27], were addressed to explore the association between HCC and a range of imaging-derived variables. Consequently, we conducted multivariate logistic regression analysis to quantify the association between imaging scores and HCC. Stepwise selection was performed using the Akaike Information Criterion (AIC) as the stopping rule to identify a parsimonious model. Model calibration was evaluated via residual deviance and overall model fit by the final AIC value. Odds Ratios (ORs) and associated 95% confidence intervals (CIs) were estimated, and statistical significance was assessed using Wald tests. Diagnostic performance of the final logistic regression model was assessed by calculating the area under the receiver operating characteristic (ROC) curve (AUC) with 95% CIs. ROC analyses were also conducted separately for each predictor to compare their discriminative ability. Optimal probability cut-offs for HCC classification were determined using the Youden index. Sensitivity and specificity were reported at this threshold. The association between model-based classification and actual HCC status was tested using the Fisher’s exact test, and corresponding ORs with exact 95% CIs were calculated. Comparative performance of predictive models was evaluated using DeLong’s test for correlated ROC curves to determine whether the combined model offered statistically superior discrimination compared to models using LVCA or LSVR alone. All analyses were performed with R software version 4.1.2 (R Foundation for Statistical Computing) and JASP software version 0.19.3.0 (JASP Team) [28], and a two-sided *p*-value < 0.05 was considered statistically significant.

## 3. Results

Considering our cohort, the most common etiology of LC was alcohol (*n* = 17, 47%), followed by dysmetabolic (*n* = 8, 22%) and infective causes (*n* = 8, 22%), including HBV and HCV infections, each accounting for *n* = 4 (11%) cases, as reported in Table 1. Autoimmune, cryptogenic, and mixed etiologies were less frequently observed. A high proportion of patients (*n* = 33, 92%) presented with at least one cirrhosis-related complication, with a mean of 2 ± 1 complications per patient. Ascites was the most common complication (*n* = 26, 72%), followed by hepatic encephalopathy (*n* = 19, 53%), portal hypertensive gastropathy (*n* = 17, 47%), and splenomegaly (*n* = 22, 61%). Esophageal and gastric varices were identified in a subset of patients: *n* = 12 (33%) had grade F1 esophageal varices, *n* = 7 (19%) had F2, and *n* = 1 (3%) had F3. Likewise, gastric varices were observed in *n* = 4 patients (11%). At the same time, hepatorenal syndrome was present in *n* = 5 patients (14%), portal vein ectasia in *n* = 4 (11%), and portal vein thrombosis in *n* = 3 (8%). Regarding the tumor characteristics, monofocal HCC was present in *n* = 10 patients (28%), while multifocal HCC was identified in only 1 patient (3%). The mean size of the hepatic nodules was 4 ± 2 cm. About the laboratory findings, the mean level of serum albumin was 4 ± 0.5 g/dL, and the mean ALT and AST levels were 38 ± 61 U/L and 48 ± 44 U/L, respectively. ALP averaged 114 ± 54 U/L, and GGT 88 ± 86 U/L. AFP levels showed a high degree of variability, with a mean of 232 ± 1356 ng/mL. Coagulation parameters showed a mean PT of 14 ± 3 s, aPTT of 36 ± 10 s, and INR of 1.3 ± 0.4. Mean platelet count was 125 ± 66 × 10^3^/μL. Serum creatinine averaged 1 ± 0.3 mg/dL. Electrolyte levels were within normal ranges, with mean potassium and sodium levels of 4 ± 0.5 mmol/L and 138 ± 4 mmol/L, respectively. Mean total bilirubin was 1.4 ± 0.9 mg/dL, and direct bilirubin was 0.7 ± 0.5 mg/dL. Inflammatory and metabolic biomarkers showed mean leukocyte, neutrophil, and lymphocyte counts of 5 ± 2, 3 ± 1.5, and 1 ± 0.5 × 10^9^/L, respectively. Mean glycemia was 120 ± 42 mg/dL, triglycerides 109 ± 64 mg/dL, total cholesterol 132 ± 38 mg/dL, HDL 42 ± 17 mg/dL, and LDL 76 ± 32 mg/dL. Regarding liver disease severity scores, *n* = 12 patients (33%) were classified as Child–Pugh class A, *n* = 21 (58%) as class B, and *n* = 3 (8%) as class C. The mean MELD and MELD-Na scores were 12 ± 4 and 11 ± 6, respectively. Finally, the mean liver volume was 1500 ± 335 cm^3^, the mean spleen volume was 685 ± 437 cm^3^, the liver right-lobe diameter averaged 15 ± 1.5 cm, and the spleen diameter was 14 ± 3 cm. In this way, the LSVR was 0.5 ± 0.3, and the LVCA was 2 ± 1.

Subsequently, we stratified the enrolled patients in two different groups based on the presence of LC or HCC (see Table 2). The mean age was comparable between the LC and HCC groups (63 ± 12 vs. 61 ± 10 years; *p* = 0.535), with a similar proportion of male patients (*n* = 18, 72% vs. *n* = 7, 64%; *p* = 0.209). Alcohol-related liver disease was the most prevalent etiology in both groups (*n* = 12, 48% in LC vs. *n* = 5, 45% in HCC; *p* = 0.888), but a significantly higher prevalence of HBV-related cirrhosis was observed in the HCC group (*n* = 3, 26% vs. *n* = 1, 4%; *p* = 0.041), while other etiologies, such as autoimmune, cryptogenic, dysmetabolic, and HCV-related disease, did not significantly differ between groups. Although complications were highly prevalent in both groups (*n* = 22, 88% in LC vs. *n* = 11, 100% in HCC; *p* = 0.230), the number of complications tended to be higher among patients with HCC (3 ± 1 vs. 2 ± 1; *p* = 0.068). Notably, esophageal and gastric varices were significantly more common in the HCC group (*n* = 4, 36% vs. *n* = 1, 4%; *p* = 0.010). Specifically, grade F2 esophageal varices (*n* = 5, 45% vs. *n* = 2, 8%; *p* = 0.009) and gastric varices (*n* = 3, 27% vs. *n* = 1, 4%; *p* = 0.041) were more frequent in the HCC group. Other complications, such as ascites, hepatic encephalopathy, portal vein thrombosis, and splenomegaly, were not significantly different between groups. Regarding laboratory measurements, AFP levels were significantly elevated in the HCC group (750 ± 2452 ng/mL vs. 4 ± 3 ng/mL; *p* = 0.013). Although most liver function parameters, including transaminases, bilirubin, and coagulation profiles, were similar between groups, platelet counts were lower in the HCC group (91 ± 40 vs. 140 ± 71 × 10^3^/μL; *p* = 0.054), approaching statistical significance. Peripheral blood analysis revealed significantly lower lymphocyte (0.9 ± 0.5 vs. 1.3 ± 0.5 × 10^9^/L; *p* = 0.030), leukocyte (4 ± 1.9 vs. 6 ± 1.6 × 10^9^/L; *p* = 0.024), and monocyte (0.3 ± 0.1 vs. 0.4 ± 0.2 × 10^9^/L; *p* = 0.026) counts in HCC patients. Moreover, total cholesterol (113 ± 23 vs. 140 ± 40 mg/dL; *p* = 0.043) and LDL (60 ± 26 vs. 84 ± 32 mg/dL; *p* = 0.035) levels were significantly lower in the HCC group. No significant differences were observed in the Child–Pugh classification (*p* = 0.435), MELD score (13 ± 4 vs. 11 ± 4; *p* = 0.204), or MELD Na score (13 ± 5 vs. 10 ± 6; *p* = 0.133) among the two different groups evaluated. CT-based imaging metrics revealed significant differences between groups. Specifically, spleen volume (1088 ± 552 vs. 507 ± 211 cm^3^; *p* < 0.001) and spleen diameter (17 ± 3 vs. 12 ± 2 cm; *p* < 0.001) were significantly higher in HCC patients. In this regard, the LSVR was significantly elevated in the HCC group (0.7 ± 0.3 vs. 0.4 ± 0.2; *p* < 0.001), as was the LVCA (3 ± 0.8 vs. 1.3 ± 0.5; *p* < 0.001).

**Figure 2:**
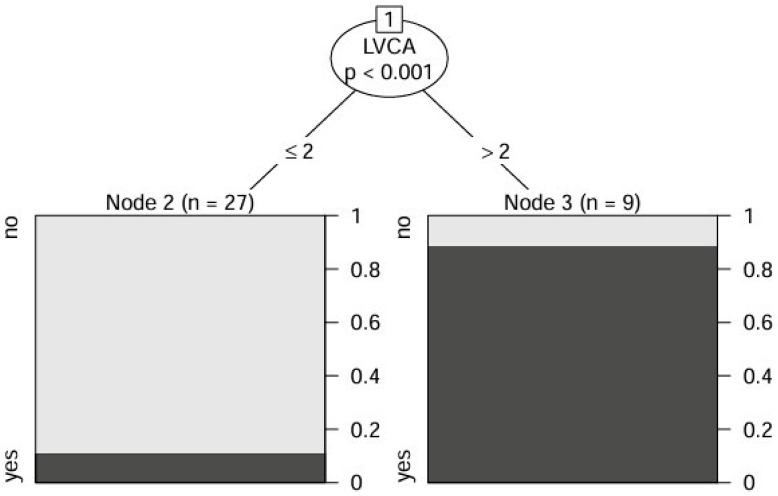
Conditional inference tree model for the prediction of HCC. The tree was constructed including LVCA, LSVR, age, and gender as input variables. Among these, only LVCA was identified as the only significant predictor and used to split the cohort into two distinct subgroups. The model indicates that patients with LVCA > 2 (right branch) had a markedly higher likelihood of HCC (8/9 = 89%), while those with LVCA ≤ 2 (left branch) exhibited a substantially lower risk (3/27 = 11%). No further splits involving other covariates were observed, highlighting the strong discriminative role of LVCA in this decision tree framework. Node sizes reflect the number of patients classified within each terminal group.

Conditional inference tree analysis was performed to explore the relationship between imaging-derived variables and the presence of HCC. Among the covariates included in the model (LVCA, LSVR, age, and gender), only LVCA was retained as a statistically significant discriminator. The resulting tree consisted of a single split, based on an LVCA threshold of 2: patients with LVCA > 2 (*n* = 9) were classified into a node with a markedly higher probability of HCC, whereas those with LVCA ≤ 2 (*n* = 27) showed a substantially lower incidence of HCC (Figure 2). Neither LSVR nor demographic variables contributed to further stratification in the tree structure, underscoring the dominant role of LVCA as an imaging predictor in this exploratory analysis.

**Figure 3:**
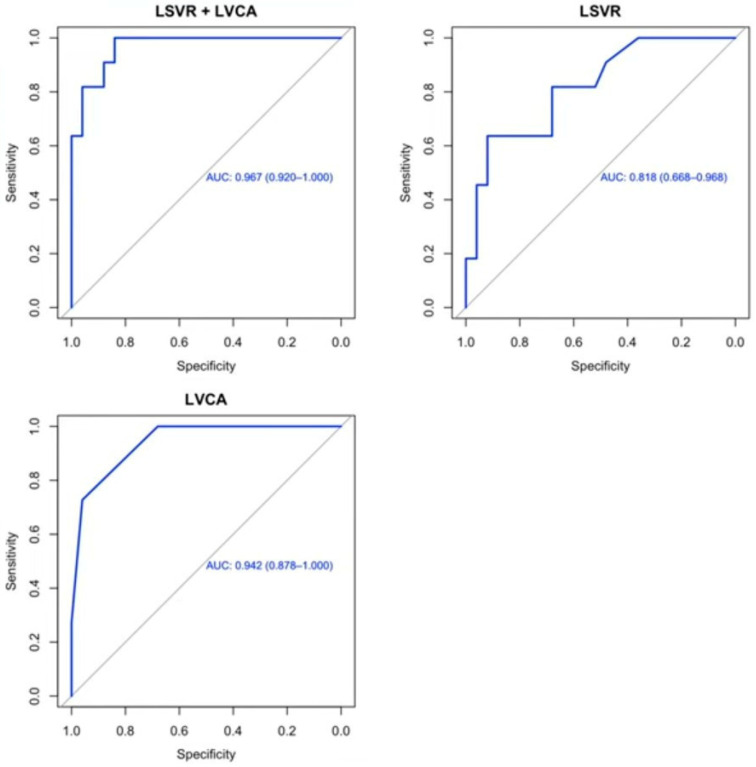
ROC curves of LSVR, LVCA, and the combined model (LSVR + LVCA) for the prediction of HCC. The top-left panel illustrates the ROC curve for the logistic regression model combining both LSVR and LVCA (combined model), achieving an AUC = 0.967 (95% CI: 0.92–1.00), indicating a higher discriminatory performance. The optimal cut-off probability for HCC prediction, identified using the Euclidean criterion, was 0.22, corresponding to a sensitivity of 91% and a specificity of 88%. At this threshold, the Fisher’s exact test confirmed a highly significant association with HCC (*p* < 0.0001, OR = 59.0). The bottom-left panel displays the ROC curve for LVCA alone, which showed an AUC = 0.942 (95% CI: 0.88–1.00), suggesting it is a strong standalone predictor. The bottom-right panel shows the ROC curve for LSVR, which yielded a lower AUC = 0.818 (95% CI: 0.67–0.97), indicating poor accuracy.

Logistic regression analysis was conducted to identify independent predictors of HCC. In the full multivariate model, including LSVR, LVCA, age, and gender, LVCA emerged as the only statistically significant predictor of HCC (OR = 3.19, *p* = 0.018). A stepwise model selection based on the AIC retained LVCA and LSVR in the final reduced model, with LVCA remaining significant (OR = 2.88, *p* = 0.0075), while LSVR exhibited a non-significant association (OR = 3.72, *p* = 0.161). Despite not reaching statistical significance, LSVR was maintained in the final model to reflect its potential additive value in combination with LVCA. Given the pilot nature of this study and the limited sample size, we opted for a cautious approach, favoring model interpretability and hypothesis generation over strict statistical parsimony. The combined model demonstrated a good overall fit (residual deviance = 16.24; AIC = 22.24), supporting its potential utility for further validation in larger cohorts. The predicted probability of HCC derived from the final logistic model yielded an AUC of 0.967 (95% CI: 0.920–1.000) using LSVR + LVCA. The individual predictors also showed good diagnostic accuracy: AUC = 0.942 (95% CI: 0.878–1.000) for LVCA and AUC = 0.818 (95% CI: 0.668–0.968) for LSVR. Using the optimal cut-off (LSVR + LVCA > 0.221), the model achieved a sensitivity of 91% and specificity of 88%, as reported in Figure 3.

The association between predicted classification and actual HCC status was statistically significant, according to Fisher’s exact test (*p* = 1.1 × 10^−5^, OR = 59.03, 95% CI: 5.65–3229.82). Finally, a comparison of AUCs using DeLong’s test showed that the combined model significantly outperformed LSVR alone (95% CI: −0.283–−0.014; *p* = 0.030), while the difference with LVCA alone was not statistically significant (95% CI: −0.059–0.008; *p* = 0.146).

## 4. Discussion

HCC is the most common primary liver cancer and a leading cause of cancer-related mortality worldwide. Its burden continues to rise, particularly in patients with underlying cirrhosis from viral hepatitis, alcohol use, or metabolic liver disease [1,3]. HCC pathogenesis involves chronic hepatic inflammation, progressive fibrosis, and cirrhosis, culminating in genomic instability and hepatocarcinogenesis [29]. Timely diagnosis of HCC is crucial, as it significantly improves patient management and long-term survival [30]. In recent years, liver transplant eligibility criteria for HCC have evolved beyond the traditional Milan criteria, incorporating dynamic predictors, such as tumor biology, response to treatment, and non-invasive biomarkers, like AFP levels [31]. According to the latest published international guidelines, transplant selection is shifting toward a more personalized, risk-adapted approach that considers tumor behavior and recurrence risk rather than size and number alone [32]. This broader perspective may expand transplant access to select patients previously deemed ineligible, potentially increasing transplant volume. However, improved surveillance, earlier detection, and novel systemic therapies may reduce the overall need for transplantation by offering effective alternatives in early or intermediate stages. As a result, transplant prioritization may increasingly focus on patients with treatment-resistant or recurrent disease, reinforcing the need for refined risk stratification tools and multidisciplinary decision-making [32].

### 4.1. Comparison Between Groups

In the present pilot study, we evaluated clinical, biochemical, and radiological parameters in patients with LC and HCC. The most prevalent etiology of LC was alcohol-related liver disease, followed by dysmetabolic and infective causes, findings that align with the existing epidemiological data in European populations [11,33,34]. While most baseline characteristics and complications were similarly distributed between groups, HBV-related LC was significantly more frequent in patients with HCC, consistent with the prior evidence highlighting HBV infection as a major risk factor for hepatocarcinogenesis [35]. Although most liver function parameters did not differ significantly, AFP levels were significantly elevated in the HCC group, supporting its utility as a traditional biomarker of HCC despite its known limitations in sensitivity and specificity [36]. Notably, inflammatory cell counts, including lymphocytes, leukocytes, and monocytes were significantly lower in patients with HCC, consistent with an immune evasion phenotype observed in malignancy [37]. Similarly, lower LDL and total cholesterol levels in HCC patients reflect prior findings linking hepatic dysfunction and malignancy to alterations in lipid metabolism [38].

### 4.2. Combined Model for the Diagnosis of HCC

Our analysis provides novel insights into the utility of CT-derived scores, particularly the LSVR and LVCA, to diagnose HCC in a cohort of patients with LC. The LSVR was significantly elevated in patients with HCC compared to those with LC. This finding is consistent with prior studies demonstrating that HCC is often associated with a preserved or even increased liver volume in early to intermediate stages, while spleen enlargement continues to reflect underlying portal hypertension [39]. In this regard, Kwon et al. showed how LSVR can predict hepatic decompensation and transplantation-free survival in patients with HBV-compensated cirrhosis, suggesting its role as potential prognostic score [24]. At the same time, LSVR showed an AUC = 0.891 as a predictor of poor native liver prognosis [40]. Regarding its potential association with other complications, this score was independently associated with clinically significant portal hypertension in patients with compensated LC and HCC [41]. LVCA, defined as a semi-quantitative score of parenchymal enhancement during contrast-enhanced CT, was significantly associated with HCC, and demonstrated a high discriminative ability in our investigation. This is biologically plausible, as HCC is characterized by neoangiogenesis and arterial hypervascularity, which enhance contrast uptake during the arterial phase [42]. The LVCA score, although relatively simple to calculate, captures these hemodynamic alterations and may serve as a surrogate for tumor-related vascular remodeling. In this context, a recent proof-of-concept study showed that LVCA used in combination with other imaging tools significantly improved the detection of clinically significant liver fibrosis in a cohort of patients with chronic liver diseases [25]. Importantly, in our study, the combined use of LSVR and LVCA improved the diagnostic performance over either parameter alone, yielding an AUC = 0.967 and a sensitivity and specificity score of 91% and 88%, respectively, for detecting HCC. Moreover, DeLong’s test confirmed that the combined model significantly outperformed LSVR alone, further reinforcing the added value of vascular imaging metrics. Regarding the use of classic biomarkers in the diagnosis of HCC, Zhang et al. conducted a large, prospective cohort study in China involving over 18,000 HBV patients, showing that AFP alone had a sensitivity of only 60% for detecting HCC, particularly lacking in early-stage disease [43] At the same time, Lok et al. evaluated AFP, DCP, and AFP-L3 in a ultrasound-based cohort and found that while DCP improved sensitivity (up to 74%), specificity remained suboptimal, and none of the biomarkers performed reliably across all tumor stages [44]. Subsequently, Tzartzeva et al. performed a meta-analysis of 32 studies, reporting that ultrasound alone had a sensitivity of 84% for detecting any-stage HCC, but only 47% for early-stage HCC, and that the addition of AFP increased early detection sensitivity to 63%, still leaving a significant diagnostic gap [45]. These studies underscore the limited utility of classical biomarkers in reliably detecting HCC, especially at early stages, and justify the need for improved imaging or combined approaches. In this regard, circulating tumor DNA (ctDNA) has been identified as a predictor of recurrence after resection in a cohort of 47 patients, 26% of whom experienced recurrence during a median follow-up of 27 months [46]. With the growing emphasis on quantitative imaging and deep learning, ctDNA is expected to become a key component alongside imaging data in radiogenomic assessments and risk stratification. This integration will likely prompt revisions in diagnostic reporting standards and classification systems [47]. From a clinical point of view, CT imaging is widely accessible and frequently performed in patients with chronic liver disease, especially when MRI is contraindicated [48,49]. The LSVR and LVCA can be derived retrospectively from routine scans without the need for specialized software, making them feasible for real-world implementation. Their integration into radiological reporting or artificial intelligence-based diagnostic tools could enhance HCC detection, facilitate risk stratification, and guide decisions regarding surveillance or liver biopsy [49]. In patients with altered levels of LVCA and LSVR, heightened clinical vigilance may be warranted even in the absence of overt radiological lesions.

### 4.3. Limitations

The main limitation of this study is that the proposed score relies on second-level diagnostic tools, such as contrast-enhanced abdominal CT scans. As a result, it is not suitable for use in screening programs aimed at the early detection of HCC. Nevertheless, it offers a valuable starting point. This pilot study lays the groundwork for future research focusing on identifying simpler and more easily measurable parameters using first-line diagnostic methods. Notably, the score highlighted that patients with HCC tend to have a larger splenic area compared to those without the disease. In our cohort, we observed a statistically significant difference in splenic size between patients with and without HCC. Since splenic diameter can be easily measured by ultrasound, the preferred screening tool for HCC in patients with cirrhosis, this finding opens the door for future investigations. Our future aim is to carry out larger studies using accessible biomarkers to enhance and simplify HCC screening, to facilitate the early detection of suspicious nodules with potential malignancy evolution. Another limitation of our study is that some patients were referred to our Center when their tumors and underlying liver disease were already at advanced stages. This may have affected the results, making it more difficult to develop a reliable score for timely tumor detection. At the same time, the small sample size limits the generalizability of our findings and may have reduced the power to detect additional diagnostic tools. Finally, the single-center, retrospective design introduces potential biases regarding patient selection.

## 5. Conclusions

Our pilot study suggests the utility of LVCA and LSVR as potential non-invasive imaging tools for HCC diagnosis. In this context, the final model incorporating both LVCA and LSVR achieved excellent discrimination. The combined model outperformed the LSVR alone, though not LVCA alone. However, new evaluations performed on a large multicenter cohort of patients are needed to confirm these findings.

## Figures and Tables

**Table 1:** Baseline characteristics of the patients studied.

	LC + HCC (*n* = 36)
**Demographic data**	
Age (years), mean ± SD	62 ± 11
Male gender, n (%)	28 (78)
**Clinical data, n (%)**	
Alcoholic	17 (47)
Autoimmune	1 (3)
Cryptogenic	3 (8)
Dysmetabolic	8 (22)
Infective	8 (22)
HBV-related	4 (11)
HCV-related	4 (11)
Mixed	1 (3)
Presence of complications	33 (92)
Number of complications (mean ± SD)	2 ± 1
Ascites	26 (72)
Hepatic encephalopathy	19 (53)
Esophageal and gastric varices	5 (14)
Esophageal varices F1	12 (33)
Esophageal varices F2	7 (19)
Esophageal varices F3	1 (3)
Gastric varices	4 (11)
Hepato-renal syndrome	5 (14)
Portal hypertensive gastropathy	17 (47)
Portal vein ectasia	4 (11)
Portal vein thrombosis	3 (8)
Splenomegaly	22 (61)
Monofocal HCC	10 (28)
Multifocal HCC	1 (3)
Size of the nodules (cm), mean ± SD	4 ± 2
**Laboratory parameters and scores, mean ± SD**	
Albumin (g/dL)	4 ± 0.5
ALP (UI/L)	114 ± 54
AST (UI/L)	48 ± 44
ALT (UI/L)	38 ± 61
GGT (UI/L)	88 ± 86
AFP (ng/mL)	232 ± 1356
Platelets (10^3^/μL)	125 ± 66
PT (s)	14 ± 3
aPTT (s)	36 ± 10
INR	1.3 ± 0.4
Fibrinogen (mg/dL)	269 ± 95
Creatinine (mg/dL)	1 ± 0.3
Potassium (mmol/L)	4 ± 0.5
Sodium (mmol/L)	138 ± 4
Total bilirubin (mg/dL)	1.4 ± 0.9
Direct bilirubin (mg/dL)	0.7 ± 0.5
Neutrophils (10^9^/L)	3 ± 1.5
Lymphocytes (10^9^/L)	1 ± 0.5
Leucocytes (10^9^/L)	5 ± 2
Monocytes (10^9^/L)	0.4 ± 0.2
Basophils (10^9^/L)	0.02 ± 0.01
Triglycerides (mg/dL)	109 ± 64
Glycemia (mg/dL)	120 ± 42
Total cholesterol (mg/dL)	132 ± 38
HDL (mg/dL)	42 ± 17
LDL (mg/dL)	76 ± 32
Child–Pugh A, n (%)	12 (33)
Child–Pugh B, n (%)	21 (58)
Child–Pugh C, n (%)	3 (8)
MELD score,	12 ± 4
MELD Na	11 ± 6
Liver volume (cm^3^)	1500 ± 335
Spleen volume (cm^3^)	685 ± 437
LSVR	0.5 ± 0.3
Liver right-lobe diameter (cm)	15 ± 1.5
Spleen diameter (cm)	14 ± 3
LVCA	2 ± 1

**Abbreviations**: LC, liver cirrhosis; HCC, hepatocellular carcinoma; HBV, hepatitis B virus; HCV, hepatitis C virus; ALP, alkaline phosphatase; AST, aspartate aminotransferase; ALT, alanine aminotransferase; GGT, γ-glutamyl transferase; AFP, alpha-fetoprotein; PT, prothrombin time; aPTT, activated partial thromboplastin time; INR, international normalized ratio; MELD, Model for End-Stage Liver Disease; LSVR, Liver to Spleen Volume Ratio; LVCA, Liver Vein to Cava Attenuation.

**Table 2:** Comparisons of patients’ characteristics based on the diagnosis of LC or LC + HCC.

	LC (*n* = 25)	LC + HCC (*n* = 11)	*p*-Value
**Demographic data**			
Age (years), mean ± SD	63 ± 12	61 ± 10	0.535
Male gender, n (%)	18 (72)	7 (64)	0.209
**Clinical data, n (%)**			
Alcoholic	12 (48)	5 (45)	0.888
Autoimmune	1 (4)	0	0.501
Cryptogenic	2 (8)	1 (9)	0.913
Dysmetabolic	7 (28)	1 (9)	0.209
Infective	4 (16)	4 (36)	0.176
HBV-related	1 (4)	3 (26)	0.041
HCV-related	3 (12)	1 (9)	0.798
Mixed	1 (4)	0	0.501
Presence of complications	22 (88)	11 (100)	0.230
Number of complications (mean ± SD)	2 ± 1	3 ± 1	0.068
Ascites	17 (68)	9 (81)	0.394
Hepatic encephalopathy	13 (52)	6 (54)	0.888
Esophageal and gastric varices	1 (4)	4 (36)	0.010
Esophageal varices F1	10 (40)	2 (18)	0.201
Esophageal varices F2	2 (8)	5 (45)	0.009
Esophageal varices F3	1 (4)	0	0.501
Gastric varices	1 (4)	3 (27)	0.041
Hepato-renal syndrome	3 (12)	2 (18)	0.621
Portal hypertensive gastropathy	11 (44)	6 (54)	0.559
Portal vein ectasia	8 (32)	4 (36)	0.798
Portal vein thrombosis	1 (4)	2 (18)	0.156
Splenomegaly	16 (64)	6 (54)	0.592
**Laboratory parameters and scores, mean ± SD**			
Albumin (g/dL)	3.8 ± 0.4	3.6 ± 0.7	0.239
ALP (UI/L)	111 ± 59	121 ± 41	0.611
AST (UI/L)	48 ± 51	47 ± 23	0.372
ALT (UI/L)	42 ± 73	29 ± 16	0.986
GGT (UI/L)	87 ± 97	90 ± 61	0.410
AFP (ng/mL)	4 ± 3	750 ± 2452	0.013
Platelets (10^3^/μL)	140 ± 71	91 ± 40	0.054
PT (s)	14 ± 3	15 ± 3	0.249
aPTT (s)	35 ± 9	38 ± 11	0.352
INR	1.3 ± 0.5	1.4 ± 0.2	0.126
Fibrinogen (mg/dL)	282 ± 98	238 ± 85	0.223
Creatinine (mg/dL)	0.9 ± 0.3	1 ± 0.4	0.409
Potassium (mmol/L)	4 ± 0.6	4 ± 05	0.334
Sodium (mmol/L)	138 ± 4	137 ± 3	0.277
Total bilirubin (mg/dL)	1.4 ± 1	1.5 ± 1	0.503
Direct bilirubin (mg/dL)	0.6 ± 0.5	0.7 ± 0.4	0.488
Neutrophils (10^9^/L)	4 ± 1.3	3 ± 1.8	0.106
Lymphocytes (10^9^/L)	1.3 ± 0.5	0.9 ± 0.5	0.030
Leucocytes (10^9^/L)	6 ± 1.6	4 ± 1.9	0.024
Monocytes (10^9^/L)	0.4 ± 0.2	0.3 ± 0.1	0.026
Basophils (10^9^/L)	0.02 ± 0.01	0.01 ± 0.01	0.165
Triglycerides (mg/dL)	121 ± 74	83 ± 21	0.057
Glycemia (mg/dL)	113 ± 29	134 ± 62	0.594
Total cholesterol (mg/dL)	140 ± 40	113 ± 23	0.043
HDL (mg/dL)	44 ± 18	38 ± 17	0.341
LDL (mg/dL)	84 ± 32	60 ± 26	0.035
Child–Pugh A, n (%)	10 (40)	2 (18)	0.435
Child–Pugh B, n (%)	13 (52)	8 (72)	
Child–Pugh C, n (%)	2 (8)	1 (9)	
MELD score,	11 ± 4	13 ± 4	0.204
MELD Na	10 ± 6	13 ± 5	0.133
Liver volume (cm^3^)	1461 ± 337	1585 ± 328	0.314
Spleen volume (cm^3^)	507 ± 211	1088 ± 552	<0.001
LSVR	0.4 ± 0.2	0.7 ± 0.3	<0.001
Liver right lobe diameter (cm)	15 ± 1.5	15 ± 1.5	0.694
Spleen diameter (cm)	12 ± 2	17 ± 3	<0.001
LVCA	1.3 ± 0.5	3 ± 0.8	<0.001

**Abbreviations**: LC, liver cirrhosis; HCC, hepatocellular carcinoma; HBV, hepatitis B virus; HCV, hepatitis C virus; ALP, alkaline phosphatase; AST, aspartate aminotransferase; ALT, alanine aminotransferase; GGT, γ-glutamyl transferase; AFP, alpha-fetoprotein; PT, prothrombin time; aPTT, activated partial thromboplastin time; INR, international normalized ratio; MELD, Model for End-Stage Liver Disease; LSVR, Liver to Spleen Volume Ratio; LVCA, Liver Vein to Cava Attenuation.

## Data Availability

The original contributions presented in this study are included in the article; further inquiries can be directed to the corresponding authors.

## Abbreviations

The following abbreviations are used in this manuscript:

AFP	Alpha-fetoprotein
AFP-L3	Lens Culinaris Agglutinin-Reactive AFP
AIC	Akaike Information Criterion
ALP	Alkaline Phosphatase
ALT	Alanine Aminotransferase
aPTT	Activated Partial Thromboplastin Time
AST	Aspartate Aminotransferase
AUC	Area Under the Curve
CI	Confidence Interval
CT	Computed Tomography
ctDNA	Circulating Tumor DNA
DCP	Des-gamma-carboxyprothrombin
GGT	Gamma-glutamyl Transferase
HBV	Hepatitis B Virus
HCC	Hepatocellular Carcinoma
HCV	Hepatitis C Virus
INR	International Normalized Ratio
LC	Liver Cirrhosis
LDL	Low-Density Lipoprotein
LSVR	Liver to Spleen Volume Ratio
LVCA	Liver Vein to Cava Attenuation
MELD	Model for End-Stage Liver Disease
MRI	Magnetic Resonance Imaging
OR	Odds Ratio
PT	Prothrombin Time
ROC	Receiver Operating Characteristic
SD	Standard Deviation
